# *Plasmodium vivax* cerebral malaria in an adult patient in Sudan

**DOI:** 10.1186/s12936-019-2961-1

**Published:** 2019-09-18

**Authors:** Maowia M. Mukhtar, Omer A. Eisawi, Seth A. Amanfo, Elwaleed M. Elamin, Zeinab S. Imam, Faiza M. Osman, Manasik E. Hamed

**Affiliations:** 1 Bioscience Research Institute, Ibn Sina University, P.O. Box 11463, Khartoum, Sudan; 2 Department of Medicine, Aliaa‘ Specialized Hospital, Khartoum, Sudan; 3 NIHR Global Health Research Unit Tackling Infections to Benefit Africa at the University of Edinburgh, Usher Institute, University of Edinburgh, Edinburgh, UK; 4 Institute of Endemic Diseases, University of Khartoum, Khartoum, Sudan

**Keywords:** *Plasmodium vivax*, Cerebral malaria, Sudan

## Abstract

**Background:**

*Plasmodium vivax* infection is rising in sub-Saharan Africa, where *Plasmodium falciparum* is responsible for more than 90% of malaria cases. While *P. vivax* is identified as a major cause of severe and cerebral malaria in South east Asia, the Pacific and South America, most of the severe and cerebral cases in Africa were attributed to *P. falciparum*. Cases of severe malaria due to *P. vivax* are emerging in Africa. A few severe *P. vivax* cases were reported in Eastern Sudan and they were underestimated due to the lack of accurate diagnosis, low parasitaemia and seldom use of rapid diagnostic tests (RDTs).

**Case presentation:**

A 60-year-old Sudanese male presented to the Al Kuwaiti hospital in the Sudan capital Khartoum. On admission, the patient was complaining of fever (measured temperature was 38 °C), sweating, chills, vomiting and confusion in the past 2 days prior to his admission. He rapidly deteriorated into a coma state within 48 h of the admission, with significant neck stiffness. He was admitted to the intensive care unit and was suspected of meningitis. Lumbar puncture was not performed since the patient was suffering from spinal cord disc. Brain CT scan was unremarkable. Several biochemical, haematological tests, and blood film for malaria were performed. The results of the laboratory tests were within the normal range except of mild elevation of the total white blood cell count and a significant decrease in the platelets count. Malaria parasites were seen in the blood film with high parasitaemia (quantified as 3 +++). The patient was diagnosed as *P. vivax* cerebral malaria based on the positive blood film and the amplification of *P. vivax* specific 499 bp amplicon using Plasmodium multi-species multiplex Polymerase Chain Reaction (PCR). The patient was treated with quinine 10 mg/kg body weight for 10 days followed by primaquine 15 mg/days PO for 2 weeks. The symptoms subsided within 48 h and the patients was cured and released from the hospital.

**Conclusions:**

*Plasmodium vivax* is an emerging cause of cerebral malaria in adults in Sudan and should be considered in the differential diagnosis of cerebral malaria for proper management of patients.

## Background

*Plasmodium vivax* infection is geographically widely spread. In 2013, it comprised 8% of all estimated malaria cases globally, and represented 47% of cases outside sub-Saharan Africa, and < 1% of cases in the WHO African Region [1]. According to the World Health Organization (WHO), there were about 15.8 million *P. vivax* cases in 2013, with the total number of malaria deaths that could be attributed to *P. vivax* ranging between 1900 and 10,000 globally.

Severe cases and deaths due to *P. vivax* malaria were reported from all endemic regions. In 2015, severe vivax malaria was attributed to cause 16% of all malaria related mortality outside sub-Saharan Africa [2]. The risk of death from *P. vivax* malaria was estimated ranging between 0.012 and 0.063%, while the risk of severe disease was estimated between 0.29 and 0.82% [2].

*Plasmodium vivax* infection is an emerging public health problem in Sudan with an overall prevalence of 26.6% among malaria cases in different regions of the country [3]. Severe *P. vivax* cases were mostly neglected and misdiagnosed as *Plasmodium falciparum* infection or confused with other fever causing disease. There were a few reports of severe *P. vivax* malaria cases in Sudan, suggesting an underestimation of the prevalence of severe *P. vivax* infection due to the low sensitivity of the currently used blood films and the seldom use of rapid diagnostic tests (RDT) [4–6].

There is a need for accurate and rapid diagnosis of severe *P. vivax* infection for differential diagnosis form *P. falciparum* cerebral cases for effective management of patients.

## Case presentation

A 60-year-old Sudanese male presented to the Al Kuwaiti hospital in the Sudan capital Khartoum complaining of fever (measured temperature was 38 °C) associated with sweating, chills, vomiting and confusion for 2 days prior to admission. He had been diagnosed with poorly managed diabetes mellitus for 20 years, and he was on Insulin Mixtard 30/70.

On his admission, the patient did not complain of any gastrointestinal Tract (GIT), urinary or respiratory disorders, but was very ill and feverish. Within 48 h post admission he rapidly deteriorated into a coma state; Glasgow coma scale of 6 and did not react to stimuli with significant neck stiffness. He was admitted to the intensive care unit and was suspected of meningitis. Lumbar puncture was not performed since the patient had spinal cord disc prolapse. Brain CT scan was unremarkable. Several biochemical and haematological tests and blood film for malaria were performed. The renal and liver parameters were within the normal range. The patient had a fasting blood sugar of 295 mg/dl, haemoglobin of 11 g/dl, a platelet count of 43,000 cells/mm3, erythrocyte sedimentation rate (ESR) 18 mm, and a total white blood count of 13.4000 cells/mm^3^.

**Fig. 1:**
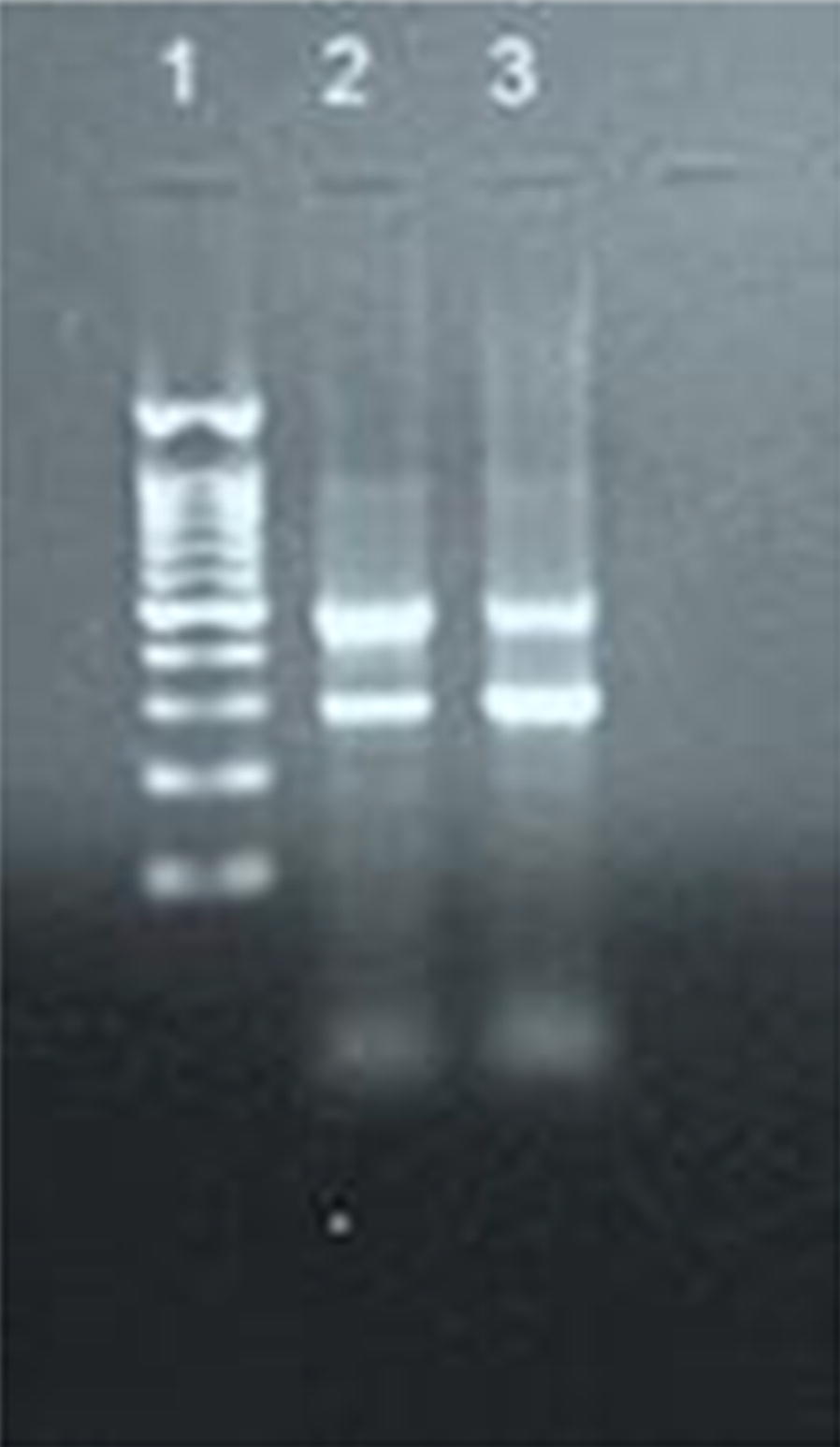
Polymerase chain reaction amplification of *Plasmodium vivax* DNA of the blood sample from the patient showing amplification of 499 bp amplicon characteristic of *P. vivax.* Lane1: Molecular weight standard 100 bp, lane 2 positive *P. vivax* control, lane 3: patient’s sample

Blood film for malaria parasites was positive by light microscopy for *P. vivax* (quantified as 3 +++). PCR amplified *P*. *vivax* specific band of 499 bp using Plasmodium multi species multiplex PCR primers (Fig. 1). The patient was diagnosed as *P. vivax* cerebral malaria case. He was started on intravenous fluids, intravenous quinine at the dose of 10 mg/kg body weight IV for 10 days and Ceftriaxone 1 g IV PD for 7 days, followed by primaquine 15 mg/days PO for 2 weeks. The vital signs improved over the next 48 h. The patient was released from the hospital 12 days post admission.

## Discussion and conclusions

The incidence of *P. vivax* infection is rising in Sudan and has increased from about 5% in 2013 to 26% of the total malaria cases [3]. Although severe *P. vivax* cases were reported from many endemic areas in South East Asia, the Pacific islands and South America, a few cases were reported in Africa reflecting the neglected status of *P. vivax* in Africa [2].

The patient described in the report was living in Khartoum the capital city of Sudan where malaria transmission is seasonal and non-endemic of *P. vivax* until recently. The fact that he was a known type 2 diabetic patients, presenting with high grade fever, chills, vomiting, sweating and confusion lead to suspicion of serious infections, including meningitis and falciparum malaria, which is the common cause of severe malaria in Sudan [5]. The rapid deterioration of the patient into coma and stiffness within 48 h of his admission increased the suspicion of meningitis and cerebral malaria and required his admission to the Intensive Care Unit (ICU). Cases of bacterial and viral meningitis were previously reported in Khartoum [6]. Lumbar puncture was recommended but the procedure was not performed since the patient had spinal cord disc making it a risky procedure. The negative finding of the CT scan excluded the possibility of central nervous system disease. The normal results of laboratory investigations including renal and liver parameters excluded renal and liver diseases. The lack of GIT and respiratory complain make the possibility of enteric infection and pulmonary TB less likely. On the other hand, the slight increase of the WBC suggested inflammatory reaction, while the low platelet counts increased the suspicion of severe infection including cerebral malaria. The finding of the blood film was decisive and demonstrated malaria parasite with high parasitaemia (+++), close examination of the thin films identified *P. vivax* infection which was confirmed by the positive result of Plasmodium multi species PCR [7]. Interestingly, most of the severe *P. vivax* malaria reported in other endemic areas including those in children in eastern Sudan were accompanied by low parasitaemia contrary to the findings of this study [8]. The fact that the patient was a known type 2 diabetes patient might had impaired his immune response leading to the increase in the parasite replication. It is worth noting that, this patient had an attack of severe *P. vivax* malaria 3 months before his current illness. He presented with fever, chills and confusion. He was successfully treated with quinine 10 mg/kg body weight IV for 10 days, ceftazidime 1G IV 8 hourly for 7 days followed by primaquine 15 mg/days PO for 2 weeks evident by the negative blood film at the end of the treatment course. The recent attack could be a recurrent attack since *P. vivax* prevails at low parasitaemia making its detection difficult [9]. It is worth noting that no previous report on *P. vivax* drug resistance in Sudan, however the possibility of noncompliance to primaquine treatment could be excluded strengthening the possibility of the persistent of the parasite from the first infection [10].

This report identified *P. vivax* as a cause of cerebral malaria in adults in Sudan and recommends considering *P. vivax* in the differential diagnosis of cerebral malaria caused by *P. falciparum* that requires a different treatment regimen. *Plasmodium vivax* treatment should be closely supervised to assure cure of the infection.

## Data Availability

Not applicable.
